# Peter’s Wife’s Mother

**Published:** 1878-12

**Authors:** 


### PETER'S WIFE'S MOTHER;

### OR, THE LONG-WINDED SERMON.

In a small town where the custom was to toll the village church bell when any of the citizens died, a clergyman announced for his text, "*And Peter's wife's mother was laid and sick of a fever.*" He found some means of expatiating on it largely, to the extent of several discourses. At length there was very decided murmuring among the townsfolk, ending in a deputation to the parson, requesting a change of subject, but it failed of its effect. And while the congregation were devising some more decided procedure, the village bell commenced tolling, to the surprise of every one, as it was not known that any one was sick; several persons went directly to the church and inquired with considerable anxiety who was dead? "*Peter's wife's mother,*" replied the sexton.

Lesson.—*Do not spin out your discourses to the little end of nothing sharpened; let them be filled with great and weighty truths, and leave it to your hearers to expatiate them in practical life.*

